# A phase I–II study of the histone deacetylase inhibitor valproic acid plus chemoimmunotherapy in patients with advanced melanoma

**DOI:** 10.1038/sj.bjc.6604817

**Published:** 2009-01-06

**Authors:** A Rocca, S Minucci, G Tosti, D Croci, F Contegno, M Ballarini, F Nolè, E Munzone, A Salmaggi, A Goldhirsch, P G Pelicci, A Testori

**Affiliations:** 1 Department of Medicine, European Institute of Oncology, Via Ripamonti 435, Milan 20141, Italy; 2 Department of Experimental Oncology, European Institute of Oncology, Via Ripamonti 435, Milan 20141, Italy; 3 University of Milan, Via Festa del Perdono 7, Milan 20100, Italy; 4 Melanoma and Muscle-Cutaneous Sarcoma Division, European Institute of Oncology, Via Ripamonti 435, Milan 20141, Italy; 5 Central Laboratory, Istituto Nazionale Neurologico ‘Carlo Besta’, Via Celoria 11, Milan 20133, Italy

**Keywords:** histone deacetylase inhibitor, epigenetic therapy, valproic acid, chemoimmunotherapy, melanoma

## Abstract

We explored in a phase I/II clinical trial the combination of valproic acid (VPA), a clinically available histone deacetylase inhibitor, with standard chemoimmunotherapy in patients with advanced melanoma, to evaluate its clinical activity, to correlate the clinical response with the biological activity of VPA and to assess toxicity. Patients were treated initially with VPA alone for 6 weeks. The inhibition of the target in non-tumour peripheral blood cells (taken as a potential surrogate marker) was measured periodically, and valproate dosing adjusted with the attempt to reach a measurable inhibition. After the treatment with valproate alone, dacarbazine plus interferon-*α* was started in combination with valproate. Twenty-nine eligible patients started taking valproate and 18 received chemoimmunotherapy and are assessable for response. We observed one complete response, two partial remissions and three disease stabilisations lasting longer than 24 weeks. With the higher valproate dosages needed to reach a measurable inhibition of the target, we observed an increase of side effects in those patients who received chemoimmunotherapy. The combination of VPA and chemoimmunotherapy did not produce results overtly superior to standard therapy in patients with advanced melanoma and toxicity was not negligible, casting some doubts on the clinical use of VPA in this setting (at least in the administration schedule adopted).

Histone acetylation is dynamically regulated by the action of histone acetylases (HATs) and deacetylases (HDACs), and it is directly involved in the regulation of gene expression: hyperacetylated histones are found at expressed genes or at genes poised for transcription, whereas hypoacetylated histones are found at silent genes and in heterochromatin (Grunstein, 1997). Recent studies have shown that HATs/HDACs may be involved in acetylation/deacetylation of non-histone proteins (including cytoplasmic factors), which may modify functional properties of the substrate (Minucci and Pelicci, 2006). Deregulation of acetylation pathways may therefore exploit multiple strategies to lead to a transformed cell phenotype, and HDAC-dependent mechanisms have been involved in the pathogenesis of multiple tumours. For this reason, HDAC inhibitors (HDACis) have been tested extensively *in vitro* and in animal models of solid tumours. The results have shown that indeed HDACis are able to induce growth arrest, differentiation and/or apoptosis of essentially all tumour cell lines tested and induce tumour regression in animal models (Saunders *et al*, 1999; Marks *et al*, 2000). These observations have triggered the beginning of clinical studies in cancer patients; in some cases (i.e., T-cell cutaneous lymphoma), the results have been extremely promising, leading to the introduction of HDACis in the clinical practice (Piekarz *et al*, 2001; Byrd *et al*, 2005).

We have identified valproic acid (VPA), a commonly used antiepileptic drug, as an HDACi (Gottlicher *et al*, 2001). Beside its neurological effects (which are not due to its HDAC inhibitory activity), VPA is able to inhibit directly, although weakly, HDACs (Ki in the millimolar range). As it has been used for decades and it is relatively ‘safe’, VPA has been therefore welcomed as a very attractive drug to enter this area, and several clinical studies have started to explore the potential of VPA as an anticancer therapy (Arce *et al*, 2006; Atmaca *et al*, 2007; Bishton *et al*, 2007; Candelaria *et al*, 2007; Munster *et al*, 2007; Rasheed *et al*, 2007, 2008).

In preclinical studies, VPA was used in murine models of acute promyelocytic leukaemia, where it caused disease remission, and in xenograft models of renal and lung metastasis, where it was highly effective in reducing the number and size of metastasis upon reinoculation of syngeneic mice with tumour cells (Gottlicher *et al*, 2001; Insinga *et al*, 2005).

Molecular evidences have been obtained to support the use of HDACis in the therapy of melanoma. Histone deacetylase inhibitors have been shown to induce strong decrease in the tumorigenic potential of melanoma-derived cell lines in animal models (xeno-transplantation), and to induce proapoptotic pathways in melanoma cells lines (Facchetti *et al*, 2004; Boyle *et al*, 2005). Strikingly, it has been suggested that resistance of melanoma cells to chemotherapy might – at least in some cases – be due to HDAC-mediated silencing of genes required for an optimal cell response to chemotherapy (such as Apaf-1) (Soengas *et al*, 2001; Soengas and Lowe, 2003), and preclinical data suggest that VPA should increase the sensitivity to chemotherapy. According to this model, critical proapoptotic pathways (silenced in cancer cells) should be reactivated following HDAC inhibition, leading to apoptosis (Minucci and Pelicci, 2006).

Additionally, HDACis have been suggested to modulate the immune response, through mechanisms which remain to be fully elucidated (Guo *et al*, 2006). In some cases, HDACi treatment has been proposed to potentiate immunoresponsive mechanisms, based on the induction of MHC molecules (Kato *et al*, 2007).

Taken together, these indications offer a rationale for the combination of HDACis with either standard chemotherapy or with immunotherapeutic approaches.

We describe the results of a phase I/II clinical trial assessing the activity of the combination of a standard chemoimmunotherapy plus VPA in patients with advanced melanoma.

## Patients and methods

Patients with histological diagnosis of locally advanced inoperable or metastatic melanoma were eligible. Other selection criteria were as follows: age >18 years and ⩽75 years, ECOG performance status 0–2, life expectancy of at least 6 months, measurable disease, adequate bone marrow function (white blood cell count >3.0 × 10^9^/l and platelet count >100 × 10^9^/l), adequate renal function (serum creatinine <120 *μ*mol l^−1^) and hepatic function (serum bilirubin < 1.5 *μ*mol l^−1^, AST <60 UI l^−1^), LDH levels ⩽2 fold the upper normal limits, written informed consent. Exclusion criteria were as follows: symptomatic cerebral or leptomeningeal involvement, other malignancy except basal or squamous carcinoma of the skin or *in situ* carcinoma of the cervix, non-malignant systemic diseases that would prevent from undergoing any of the treatment options, psychiatric or addictive disorders that would prevent from giving informed consent, pregnancy or breastfeeding, manifest severe hepatic and pancreatic dysfunction, porphyria, previous chemoimmunotherapy within 40 days.

All patients signed an institutional review board-approved informed consent form. The study was conducted in accordance with the principles of the Helsinki Declaration.

Baseline evaluation included the following: medical history, physical examination, ECOG performance status, haematology and blood chemistry, ECG, chest X-ray or CT scan of the thorax, CT scan or US of the abdomen. Target lesions were studied with CT scan at baseline and for response evaluation.

### Study design and treatment plan

The study has been originally designed as a phase II clinical trial, with treatment plan involving an induction with VPA alone, whose dosage was gradually increased in each patient until achieving biologically active concentrations (‘optimal concentration’), and then administered for 4 weeks, followed by a combined treatment with chemoimmunotherapy plus VPA. The aim of the induction phase was re-establishing the expression of genes, such as Apaf-1, that have been shown to be silenced by HDAC-involving mechanisms, and that are linked to response to chemotherapy (Soengas *et al*, 2001). Because the main aim of the trial was the evaluation of objective response to the combined treatment, although no formal assessment of response to the induction phase was planned, patients with overt disease progression during this phase were taken off study and received treatment at the discretion of the investigator.

We adopted a standard schedule of VPA administration used in epileptic patients, starting with 10 mg kg^−1^ day^−1^ (in three divided doses) and increasing the dose weekly by 10 mg kg^−1^ day^−1^ (with a maximum allowed dose of 30 mg kg^−1^ day^−1^), up to the achievement of adequate plasmatic concentrations. These doses usually allow to maintain steady-state plasma concentrations between 50 and 125 mg l^−1^ (DeVane, 2003), potentially in the range required to modulate the target (HDACs). Prudent initial doses were chosen to avoid haematological and neurological side effects that are dose related and could be more unpredictable at higher doses, due to the nonlinear metabolism/clearance and protein binding of VPA (DeVane, 2003). Furthermore, the use of these doses of VPA as anticonvulsant drug in association with chemotherapy in patients with cerebral tumours had been reported as feasible, although a slight increase in haematological toxicity is described (Bourg *et al*, 2001). Valproic acid was supplied in tablets from 200 and 500 mg, and total daily dose was rounded off to the nearest dose allowed by the available tablets, and administered in three divided doses every 8 h.

After 4 weeks of full dose VPA, patients received dacarbazine, 800 mg m^−2^ intravenously every 21 days, and interferon-*α*, 600.000 IU twice daily subcutaneously, while continuing VPA at the same dose. Treatment was planned for a maximum of four courses of dacarbazine and 6 months of interferon-*α*, or until disease progression. Antiemetic prophylaxis with serotonin antagonists and corticosteroids was used before administering dacarbazine.

**Figure 1:**
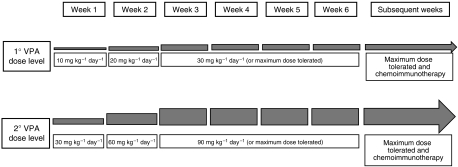
Valproic acid dose escalation scheme. See the Patients and Methods section for a more detailed description of the treatment schedule and rationale.

The analysis of VPA plasmatic levels and histone acetylation changes for the first 10 patients showed that the dose of 30 mg kg^−1^ day^−1^ did not allow either the achievement of the optimal plasmatic concentration of free drug (50–100 mg l^−1^) in most patients or a consistent increase in histone acetylation. The study design was therefore modified into a phase I/II clinical trial, with an amendment to the protocol, introducing a second dose level of maximum 90 mg kg^−1^ day^−1^ of VPA, based on plasmatic levels achieved with the 30 mg kg^−1^ day^−1^ dose and on expected toxicity (Figure 1). Seven patients who had already completed the induction phase at 30 mg kg^−1^ day^−1^ as maximum dose had weekly dose escalation by 30 mg kg^−1^ day^−1^ during the combination phase, whereas new patients started the induction phase with 30 mg kg^−1^ day^−1^ and increased weekly by 30 mg kg^−1^  day^−1^ until the achievement of the required plasmatic concentration, which was continued for 4 weeks alone and then in combination with dacarbazine and interferon-*α*.

The primary end point was the response rate with the combination of VPA plus chemoimmunotherapy. Secondary end points were the assessment of the toxicity of VPA, alone and combined with chemoimmunotherapy, the evaluation of clinical benefit (defined as the overall rate of objective response plus disease stabilisation longer than 24 weeks), response duration and progression-free survival. Histone acetylation levels were measured in peripheral blood mononuclear cells (PBMCs) to assess correlation with patient response.

### Toxicity evaluation and dose modifications

Patients were assessed for toxicity weekly during the induction with VPA alone and before every cycle of chemotherapy during the combined treatment, with clinical examination and laboratory evaluation. Toxicity was graded using the Common Toxicity Criteria (CTC) Version 2.0 (http://ctep.cancer.gov/forms/CTCv20_4-30-992.pdf), and common dose modifications or delays were adopted for chemotherapy in case of toxicity. Valproate dose was reduced (one step at a time, following the reverse of the escalation plan for each dose level) in case of grade 2 toxicity attributable to the drug, and temporarily stopped in case of grade 3–4 side effects, until resolution to at least grade 2. Treatment was then resumed with dose reduction as in case of grade 2 toxicity.

### Response assessment

Assessment of response was done before each cycle of chemotherapy by physical examination in case of superficial lesions. Computerized chemography for assessment of target lesions was done after 8 weeks of combined therapy and repeated every 8 weeks thereafter (no assessment was planned at the end of the induction period). Tumour response was defined according to RECIST criteria (Therasse *et al*, 2000).

### VPA plasma levels and histone acetylation in peripheral blood cells

**Figure 2:**
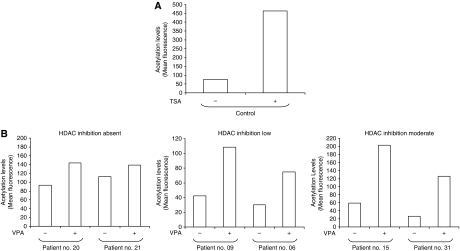
(**A**) FACS analysis of reference U937 cells not treated and treated with 50 ng ml^−1^ of trichostatin A (TSA) for 4 h. The reference cells were analysed at every measurement, to ensure consistency in machine settings and blank determination (see also Ronzoni et al, 2005).(**B**) Representative FACS analyses of patients untreated or treated for 2 weeks with VPA. Peripheral blood mononuclear cells were collected and analysed as described in the Patients and Methods section. Patients shown in the figure have been classified according to the level of increase in histone acetylation: absent (<2 fold), low (twoto three-fold), moderate (>3 fold).

Measurement of VPA plasma levels (absolute and free levels) was done at the end of the 1st week of treatment (four times daily: 0800 hours, before first daily administration; 1100 hours, 3 h post-dose; 1500 hours, 7 h post-dose; 1800 hours, 2 h after the second daily dose) and repeated at the beginning of the combination phase and weekly thereafter (at 0800 hours, before first administration) or whenever new drugs were introduced in the treatment of the patient. To define the ‘optimal concentrations’ (biologically active concentrations), we followed two criteria: (a) the plasmatic concentrations of VPA. We measured both total and free levels of the drug by FPIA method (fluorescence polarised immunoassay – Abbott Industries). The free levels were performed on plasma ultrafiltrates (Levy *et al*, 1984). Given the molecular weight and its relatively low potency (Ki in the millimolar range), we aimed to reach concentrations of free VPA in the millimolar range (50–100 mg l^−1^ or higher); (b) the levels of induction of histone acetylation in PBMCs. We used cytofluorimetric techniques to evaluate histone acetylation levels in PBMCs, which in preclinical models of acute myeloid leukaemia have been good indicators of the efficacy of VPA treatment (Ronzoni *et al*, 2005). Assessments were done at the end of the second week of treatment, at the beginning of the combination phase and weekly thereafter. The levels of histone acetylation in PBMCs from representative patients are reported in Figure 2, together with results from a cell line treated with trichostatin A as positive control.

### Statistical considerations

The main end point was the best tumour response. Using a two stage, phase II optimal design, powered to choose between an acceptable response rate of 40% and an unacceptable one of 20%, at the 5% significance level and 90% power, 19 patients were needed in the first stage and if four or less had an objective response than the trial had to be closed because of poor response. If five or more patients had a response, the trial would go on to the second stage by recruiting further 35 patients to reach a total of 54. If 16 patients or more had an objective response, it could be concluded that a response rate of 40% is possible and one of 20% unlikely.

Because the main aim was to evaluate the overall activity of the combination of VPA plus chemoimmunotherapy, the assessment of treatment activity is performed on patients actually receiving the combined treatment, but results of an intent-to-treat analysis on all eligible patients are also reported.

Time-to-event end points were estimated using the method of Kaplan and Meier. Progression-free survival was measured from the first day of study treatment to the date of disease progression. The association between paired continuous variables was assessed by Spearman's correlation coefficient. Statistical analysis was performed with R (R Development Core Team, 2006).

## Results

Between September 2001 and February 2002, 32 patients referred to the Division of Medical Oncology and to the Melanoma and Muscle-Cutaneous Sarcoma Division of the European Institute of Oncology were entered sequentially onto the study. Two patients turned out to be ineligible, one with no evidence of disease and the other because of a second malignancy. Among 30 eligible patients, 28 were assessable for toxicity, whereas one patient never started treatment because of early refusal and one never reached meaningful plasmatic levels of VPA because of malabsorption. Eighteen patients received the combined treatment with VPA and chemoimmunotherapy and were assessable for response. Ten patients were not assessable, eight for early progressive disease (before starting chemotherapy), one for non-compliance and one who was admitted to another hospital and lost to follow-up. The characteristics of all 32 registered patients are listed in Table 1. Apart from most patients being pretreated for metastatic disease, the distribution of main features is roughly comparable with that from most recent large trials of first-line biochemotherapy (Eton *et al*, 2002; Ridolfi *et al*, 2002; Bajetta *et al*, 2006).

Overall, 29 patients started the induction phase, receiving VPA alone for a median duration of 6 weeks (range 3–10 weeks), including dose escalation and 4 weeks at maximum dose. Eighteen patients went on the combination phase, receiving a median of four courses of chemotherapy (range 1–6) in association with interferon-*α*, with 11 patients completing the planned programme of four courses (one responsive patient received six courses of chemotherapy).

Table 2 illustrates the amount of VPA received by patients, reporting the maximum daily dose assumed and the median duration of treatment at the maximum dose.

Among the initial cohort of 10 patients treated at the first dose level of VPA, three did not undergo dose escalation above 10 mg kg^−1^ day^−1^, one for non-compliance and two because of disease progression. The other seven reached the maximum dose of 30 mg kg^−1^ day^−1^, but stopped treatment because of disease progression before starting chemotherapy. In the second cohort of patients, 15 reached a dose of VPA of 60 mg kg^−1^ day^−1^, without further escalation because of toxicity, and four reached the planned maximum dose of 90 mg kg^−1^ day^−1^, which was later reduced or temporarily suspended in all of them; 11 completed the planned treatment with VPA plus four courses of chemotherapy and 6 months of interferon-*α*; the other eight patients stopped treatment early, because of disease progression.

The median duration of treatment with an optimal dose of VPA was less than 1 month. Table 3 shows the toxicity registered according to the maximum dose of VPA. During the induction phase, most side effects were mild and included myelosuppression, gastrointestinal and hepatic toxicity and neurological toxicity; more serious side effects were rare, and more likely due to disease progression (see below). During the combination phase, myelosuppression was more severe. Most non-haematological side effects were mild, but neurological toxicity exceeded that seen during the induction phase.

Two patients had serious adverse events possibly related to VPA. One had grade 4 cerebral haemorrhage, headache and coma, and multiple grade 3 neurological symptoms and grade 3 hypocalcemia. Laboratory parameters, including coagulation tests, were normal. An MRI of the brain showed a massive haemorrhage in the right cerebral hemisphere; a neuroradiological consultant favored the hypothesis of bleeding of a cerebral metastasis, although the patient was not known to have cerebral metastases before entering the study. The patient was taking VPA at the dose of 60 mg kg^−1^ day^−1^; total VPA concentration was 148.9 mg l^−1^ and free VPA concentration was 51 mg l^−1^. Histone acetylation in peripheral blood leukocytes was increased 2.9 times over the baseline values. Four days after suspension, VPA total and free concentrations were dropped to 3.1 and 0.2 mg l^−1^, respectively. The patient was also receiving dacarbazine and interferon-*α*. Consciousness recovered to normal, but the patient had permanent left hemiparesis.

A second patient had neurological toxicity (grade 3 confusion, stupor, ataxia, urinary incontinence and grade 2 tremor) probably related to VPA while taking a dose of 35 mg kg^−1^ day^−1^ plus dacarbazine and interferon-*α*. Symptoms disappeared after suspension of VPA.

Three other serious adverse events were considered unrelated to the study treatment and attributed to tumour progression: one case of pleural effusion with severe dyspnoea, one case of cachexia and one case of grade 4 anaemia with haematuria due to melanoma progression involving the bladder.

The two bleeding episodes reported (one cerebral haemorrhage with combined therapy and one haematuria with VPA alone) could suggest a possible bleeding diathesis, potentially related to the interference of VPA with platelets and haemostasis, but this possibility was not supported by alterations in common coagulation tests.

The treatment produced one complete remission, two partial remissions and three disease stabilisation lasting at least 24 weeks. Twelve patients had progressive disease or short-term (2 months) stabilisation, and 12 were not evaluable for response and were considered as treatment failures. The overall response rate is thus 10% (95% CI: 2–27%) when considering all eligible patients (*n*=30), and 17% (95% CI: 4–41%) when considering only evaluable patients (*n*=18). The rate of clinical benefit, in terms of objective response or disease stabilisation lasting at least 24 weeks, is 20.7% (95% CI: 8.0–39.7%) on all eligible patients and 33.3% (95% CI: 13.3–59.0%) on evaluable patients. The median time to progression (TTP) is 3.8 months.

The study was closed early for poor activity, after the enrolment of 18 evaluable patients in the first stage, because only three responses were seen by that time, while five or more responses should have been registered among the first 19 patients to proceed to the second stage. Most patients who obtained a clinical benefit were pretreated with interferon-*α* as adjuvant (three patients) or palliative (three patients) therapy; one patient who achieved disease stabilisation was pretreated with dacarbazine for advanced disease. The TTP has been for at least 40 weeks in four patients.

**Figure 3:**
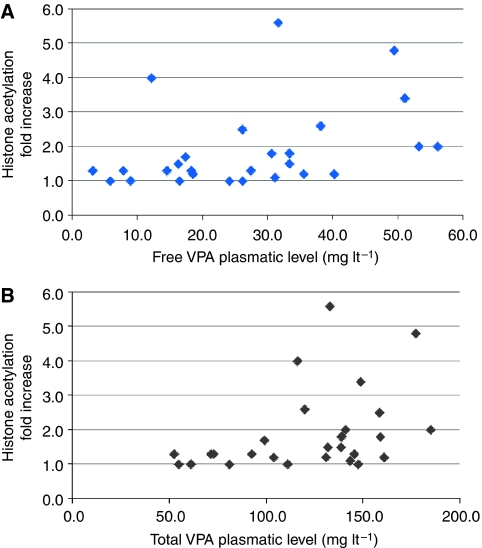
Relationship between free (**A**) and total (**B**) valproic acid (VPA) plasmatic levels and fold increase in histone acetylation in peripheral blood mononuclear cells over baseline values. Data refer to the maximum values of plasmatic VPA and the maximum fold increase in histone acetylation reached by each patient during treatment at the maximal dose of VPA. For a statistical analysis of the data, see the Results section. Note that the patients who showed the highest increase in histone acetylation levels (>4 fold) did not maintain this increase at repeated measurements, notwithstanding the maintenance of the VPA dose.

Plasmatic levels of total and free VPA were measured in 27 patients, and maximum levels reached are reported in Table 4. There is a statistically significant correlation between VPA dose and both total (Spearman's *R*=0.65, *P*=0.0002) and free (Spearman's *R*=0.63, *P*=0.0005) VPA plasmatic levels, as well as between increase in histone acetylation and both total (Spearman's *R*=0.44, *P*=0.02) and free (Spearman's *R*=0.45, *P*=0.02) VPA plasmatic levels (Figure 3). On the contrary, there was no clear association between dose and increase in histone acetylation (Spearman's *R*=0.22, *P*=0.3). There was no correlation between histone acetylation and response. All patients who achieved greater than twice increase in histone acetylation had disease progression (data not shown).

## Discussion

Stage IV melanoma has a poor prognosis, with median survival shorter than 1 year and a negligible proportion (⩽5%) of patients alive at 5 years (Lee *et al*, 2000) after chemotherapy or immunotherapy. Although dacarbazine is considered the standard therapy, a meta-analysis of randomised trials showed a response rate of 16.9% (95% CI: 14.7–19.1%) (Huncharek *et al*, 2001), and randomised phase III trials with audited response assessment yielded a response rate as low as 7% (Avril *et al*, 2004). Despite encouraging results in phase II studies, neither polychemotherapy (Chapman *et al*, 1999) nor biochemotherapy (Eton *et al*, 2002; Ridolfi *et al*, 2002; Bajetta *et al*, 2006) have shown clear advantages over single-agent dacarbazine in randomised trials. Therefore, new therapeutic strategies are clearly needed.

Histone deacetylase inhibitors are considered among the most promising new anticancer drugs, and several agents have recently entered clinical trials. The use of HDACis has been suggested also for the treatment of patients with melanoma (Boyle *et al*, 2005). The use of VPA is extremely appealing. Although not as potent as other HDACis developed more recently, this drug has been for decades in the treatment of epilepsy, with generally mild side effects reported. Valproic acid has been shown recently to be active on melanoma cell lines, further suggesting a potential use in this disease (Valentini *et al*, 2007). In our study, however, the combination of dacarbazine, interferon-*α* and VPA did not appear to be clearly superior in terms of activity to what is expected from standard therapy.

The use of VPA as HDACi was hampered by a series of problems. Valproic acid dosage must be adjusted gradually, requiring a few weeks before reaching the full dose in most patients, which may result in disease progression in aggressive tumours. Eight patients in our study had early disease progression, before starting chemotherapy. The drug turned out to be less tolerable than when used alone for treatment of epilepsy, particularly with dosages of 60 mg kg^−1^ day^−1^ or higher. The median duration of treatment with an optimal dose of VPA was less than 1 month, and most patients had dose reductions or temporary interruption of treatment for toxicity. Two serious adverse events, a grade 4 bleeding of a cerebral metastasis and a grade 3 neurological toxicity, were possibly related to the study drugs and required definitive interruption of VPA.

Preclinical studies have shown that indeed VPA inhibits efficiently HDACs, and extended survival with tumour regression in several cancer models (Insinga *et al*, 2005). A comparison of the use of VPA in mice and human patients, however, reveals several important differences. In fact, we were able to reach free plasmatic concentrations of VPA >2 mM in our mice models (>300 mg l^−1^ of free VPA) (Insinga *et al*, 2005). In contrast, only 44% of the patients showed free VPA levels >30 mg l^−1^ (equivalent to a concentration >0.2 mM) and just three patients reached a free VPA concentration >50 mg l^−1^ (>0.3 mM). These concentrations are suboptimal, and the increase in histone acetylation levels observed in PBMCs of the patients was never as high as those observed in preclinical models. In fact, in murine models of leukaemia, we were unable to achieve clinical responses (i.e., disease remission and extended survival) at free VPA levels below 1 mM (A Insinga, PG Pelicci and S Minucci, unpublished observations). At those levels, the average increase in histone acetylation levels observed in blood-derived mononuclear cells (which in leukaemic mice are a mix of leukaemic cells and PBMCs) is always >4 fold, an increase which was not observed in this clinical study. Only two patients showed a >4 fold increase in histone acetylation, but those increases were not observed throughout the entire treatment regimen, and therefore cannot be considered as true exceptions to this model. In the absence of a set of patients, which show sustained high levels of histone acetylation following treatment, however, it cannot be claimed that the relationship observed in the leukaemic murine models holds true in the clinical setting, and further studies will be required.

From our study it appears difficult to further increase VPA doses in continuous administration, without incurring in undesired, serious side effects, at least when combined with dacarbazine and interferon-*α*. Other studies have reported the feasibility and partial activity of a continuous oral treatment with lower doses of VPA of 30–40 mg kg^−1^ day^−1^ in combination with hydralazine and chemotherapy (Arce *et al*, 2006; Candelaria *et al*, 2007), and the potential to improve results of chemotherapy alone and to overcome chemotherapy resistance in solid tumours (Candelaria *et al*, 2007). It is not possible to discern the relative contribution of the two epigenetic drugs to these results, nor if the addition of hydralazine could allow for a reduction in VPA doses without jeopardising its activity. In our study, the mean plasmatic level of total VPA in patients receiving doses ⩽30 mg kg^−1^ day^−1^ was 88 mg l^−1^, producing a 1.7 mean fold increase in histone acetylation, which is considered insufficient from preclinical data. For patients receiving doses >30 mg kg^−1^ day^−1^, the mean plasmatic concentration was 136 mg l^−1^ with a two-fold mean increase in histone acetylation. Although we found a correlation between VPA dose and plasmatic levels and between plasmatic levels and histone acetylation changes, there was no significant correlation between dose and histone acetylation changes. Individual differences in VPA pharmacokinetics and pharmacodynamics, related for example to baseline values of histone acetylation and activity of HDACs, could explain the lack of a direct relationship between VPA dose and biological activity, which hampers the definition of the optimal dose of this agent. Other studies have explored cyclic schedules. With an intravenous administration of VPA daily for 5 consecutive days in 21-day cycles (Atmaca *et al*, 2007), the MTD was 60 mg kg^−1^ day^−1^ and histone hyperacetylation and HDAC downregulation were detected on PBMCs in the majority of patients. The administration of a loading dose (either intravenously or orally) of VPA followed by 5 oral doses administered every 12 h, preceding the administration of epirubicin 75–100 mg m^−2^, allowed the achievement of 140 mg kg^−1^ day^−1^ as recommended dose of VPA for phase II studies (Munster *et al*, 2007), showing a significant correlation among histone H4 acetylation and VPA dose and plasmatic concentration, as well as clinical antitumor activity.

Different administration schemes or combinations with other chemotherapic regimens could play, therefore, an important role in the determination of the response to VPA. Conflicting results have been reported about the correlation among VPA dose, plasmatic concentration and changes in histone acetylation, and more data are needed to better define these relationships and the value of these parameters as surrogate end points. The same considerations could apply to other, more potent HDACis, such as vorinostat and belinostat, which appear promising in enhancing the activity of chemotherapy regimens (Ramalingam *et al*, 2007; Sinha *et al*, 2007).

In conclusion, continuous oral treatment with VPA at doses of 60 mg kg^−1^ day^−1^ or greater seems not feasible in patients with advanced melanoma receiving concomitant dacarbazine and interferon-*α*. Administration of VPA with different schedules (e.g., cyclically in concomitance with chemotherapy, or continuously but at lower doses in concomitance with other epigenetic or biological drugs), however, could be feasible and treatment of a higher number of patients could reveal subsets of responsive patients. Further development of VPA as HDACi in patients with solid tumours requires careful consideration of the treatment schedule, synergism with other drugs and ideally the definition of predictors of response.

## Figures and Tables

**Table 1:** Patient characteristics

**Characteristic**	***N***
*Sex*	
Male	17
Female	15
	
*Age, years*	
Median	51
Range	23–72
	
*ECOG performance status*	
0	26
1	5
2	1
	
*Number of involved sites*	
0	1
1 (sc/ln)	6 (4/2)
2	9
>2	16
	
*Tumor site*	
M0	1
Skin, sc, ln	11
Lung	2
Other visceral/(CNS)	18/(2)
	
*Previous treatments*	
Surgery	31
*Adjuvant treatment*	
CT (DTIC)	1
IT (IF-*α*)	12
None	19
*Treatment for metastatic disease*	
CT (DTIC/CVD)	4 (4/1)
IT (IF-*α*)	5
CT+IT	8
DTIC o CVD+IF-*α* (±IL-2)	7
DTIC o CVD+IL-2	1
Hyperthermic isolated limb perfusion	1
None	14
*N. lines for metastatic disease*	
0/1/2/>2	14/8/6/4
Median (range)	1 (0–5)

Abbreviations: sc=subcutaneous; ln=lymph nodes; CNS=central nervous system; ECOG=Eastern Cooperative Oncology Group; CT=chemotherapy; IT=immunotherapy; IF-*α*=interferon-*α*; DTIC=dacarbazine; CVD=cisplatin vinblastine dacarbazine.

**Table 2:** Maximum dose of valproic acid (VPA) received during the entire treatment period^a^

	**Maximum VPA dose (mg kg ^−1^day^−1^)**			
	**(First cohort, *N*=10)**		**(Second cohort, *N*=19)**	
	**10**	**30**	**60**	**90**
Number of patients	3	7	15	4
Median duration at maximum dose (days)	8	16	26	19
Temporary suspension (number of patients)	1	1	4	3
Dose reduction (number of patients)	1	4	5	3
Stopped VPA in advance (number of patients)^b^	3	7	6	2

a On 29 patients who actually started VPA assumption.

b Five serious adverse events, 1 non-compliance, 12 treatment failures.

**Table 3:** Toxicity of valproic acid (VPA)^a^

				**NCI – CTC grade**			
				**1**	**2**	**3**	**4**
**VPA dose (mg kg^−1^ day^−1^)**	**No. of patients**	**Median duration (weeks)**	**Toxicity**	**(No. of patients)**	**(No. of patients)**	**(No. of patients)**	**(No. of patients)**
*VPA alone*							
10–30^b^	17	6	Leukocytosis	2			
			Thrombocytosis	1			
			Anemia	2		2	
			Nausea/vomiting	2			
			Abdominal pain		1	1	
			Asthenia		3		
			Erithema		1		
			Hepatic (ALP, LDH)		1		
			Amylase	3	1		
			Dyspnea (pleural eff.)				1
			Depr. consciousness	1			
							
60–90^c^	11	6	Thrombocytopenia	1			
			Anemia	1	1	1	1^d^
			Asthenia	1			
			Constipation	1^d^			
			Sciatic pain		1		
			Infection		2^d^		
			Creatinine	1			
			Hematuria		1^d^		
			Hypoacusis	1^d^			
			Dyspnoea	1			
			Ataxia			1	
			Depr. consciousness	1^d^	1		
							
*VPA in combination with chemoimmunotherapy*							
30	3	3	Anemia		1		
			Asthenia		2		
			Hepatic (AST)	1			
			Tremors	1			
							
60	12	14	Leukopenia	2	4		
			Neutropenia		2	3	
			Thrombocytopenia	3	1	4	
			Anemia	2	3	1	1
			Nausea/vomiting	6			
			Diarrhea	3	1		
			Asthenia		2		
			Ammonia	2			
			Hepatic (any)	3			
			Gastric pain	2			
			Fever	1	1		
			Hypocalcemia			1	
			Depr. consciousness	3	3	1	1
			Other neurological^e^	2	6	2	1
			Cerebral hemorrhage				1
			Urinary incontinence		1	1	
							
90	3	3	Leukopenia		1		
			Thrombocytopenia			1	1
			Anemia	1			
			Hepatic (AST)	1			
			Amylase	1			
			Headache		1		

a On 28 assessable patients. Each patient is reported in each section of the table (VPA alone, and VPA in combination with chemoimmunotherapy) within the row corresponding to the highest dose level he received during the pertinent period of therapy. Some patients had dose reductions of VPA already during the induction phase, and others had dose escalation during the combination phase. All patients except one had some kind of toxicity.

b Three patients at 10 mg kg^−1^ day^−1^, 14 patients at 30 mg kg^−1^ day^−1^.

c 10 patients at 60 mg kg^−1^ day^−1^, one patient at 90 mg kg^−1^ day^−1^.

d 90 mg kg^−1^ day^−1^, with bladder progression of disease.

e Vertigo, hallucinations, headache, speech impairment, mood alteration, memory loss, paraesthesia, seizures, tremors, confusion.

**Table 4:** Plasmatic levels of total and free VPA

**Total VPA plasmatic levels (mg l^−1^)**	**No. of patients**
0–50	0
51–100	8
101–150	14
151–200	5
	
**Free VPA plasmatic levels (mg l^−1^)**	**No. of patients**
⩽10	4
10.1–20	7
20.1–30	4
30.1–40	7
40.1–50	2
50.1–60	3

Abbreviation: VPA=valproic acid.
